# The complete chloroplast genome sequence of *Platanthera minor*

**DOI:** 10.1080/23802359.2019.1696241

**Published:** 2019-12-09

**Authors:** Si-Si Chen, Bi-Cai Guan

**Affiliations:** a College of Life Sciences, Nanchang University, Nanchang, China;; b State Key Laboratory of Systematic and Evolutionary Botany, Institute of Botany, Chinese Academy of Sciences, Beijing, China

**Keywords:** Orchidoideae, chloroplast genome, inverted repeats, phylogeny, *Platanthera*

## Abstract

*Platanthera minor* is widely distributed in East Asia. The complete circular chloroplast genome with a length of 154,430 bp possesses the typical structure, consisting of a large single copy (LSC) of 83,536 bp, a small single copy (SSC) of 17,612 bp, and two inverted repeats (IR) of 26,641 bp. The average GC content of the genome is 36.7%. The circular *P. minor* chloroplast genome contains 114 genes, including 80 protein-coding genes, four rRNA genes, and 30 tRNA genes. The chloroplast sequence provided a resource for analyzing genetic diversity of the Orchidaceae family.

The Orchidaceae is one of the largest plant families, which is believed to contain more than 20,000 species (Dixon et al. 2003; Chase et al. 2015). *Platanthera minor* is a member of the genus *Platanthera* belonging to the subfamily Orchidoideae, widely distributed in East Asia. *Platanthera minor* mainly grows under forest on slopes, alpine meadows, with an altitude of 200 to 3000 m. So far, only two complete chloroplast genomes from the genus *Platanthera* has been reported (Dong et al. 2018; Lallemand et al. 2019). There have been no studies on the genome of *P. minor*. In this study, we reported the complete circular chloroplast genome sequence of *P. minor* and used the sequence for phylogenetic analysis.

*Platanthera minor* used for sequencing were collected from Tengchong Yunnan, China (25°0′06.9″N, 98°19′31.5″E). The voucher specimens (JXH10398) are deposited in the Herbarium, Institute of Botany, Chinese Academy of Sciences. The dry leaves were used to extract total DNA by CTAB (Li et al. 2013), and the complete chloroplast sequence was obtained using Illumina Hiseq 2500 and assembled after trimming using Geneious (Kearse et al. 2012). The DNA was deposited in State Key Laboratory of Systematic and Evolutionary Botany, Institute of Botany, Chinese Academy of Sciences, Beijing, China. The average coverage was 484.2. This sequence was annotated using PGA (Qu et al. 2019), and we manually checked the correction.

The circular *P. minor* chloroplast genome (GenBank accession MN416689) contains 114 genes, including 80 protein-coding genes, four rRNA genes, and 30 tRNA genes. Thirteen genes contain single intron but *clpP, rps12* and *ycf3* genes have two introns. The length of the complete chloroplast of *P. minor* is 154,430 bp, composed of a large single copy (LSC) of 83,536 bp, a small single copy (SSC) of 17,612 bp, and two inverted repeats (IR) of 26,641 bp. The average GC content of chloroplast is 36.7%.

The phylogenetic tree was constructed based on the whole chloroplast genome of 13 species representing five orchid subfamilies. All protein-coding genes exported from Geneious (Kearse et al. 2012) and were aligned together with other representatives of Orchidoideae. We manually checked in BioEdit (Hall 1999) and concatenated these aligned sequences using SequenceMatrix (Vaidya et al. 2011) later. The phylogenetic analysis was performed using maximum likelihood method by IQtree with 1000 ultrafast bootstrap (Nguyen et al. 2015). The phylogenetic tree indicated that the *P. minor* is closer to *Habenaria pantlingiana* in Orchidoideae (Figure 1). Our chloroplast sequence provided a resource for analyzing the genetic diversity of the Orchidaceae family.

**Figure 1.**
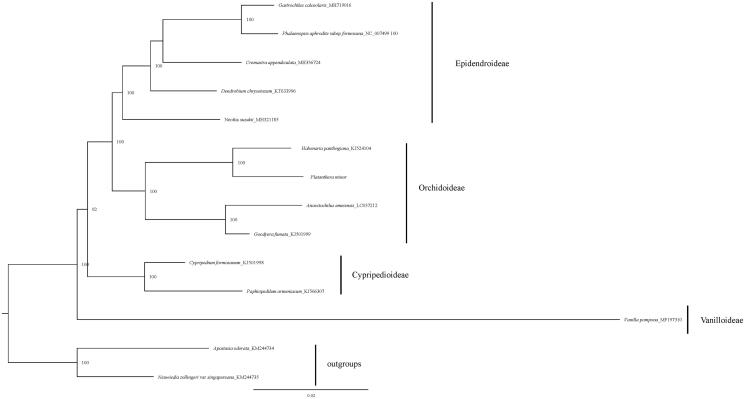
The maximum likelihood tree of *P. minor* and 13 other orchid based on whole chloroplast genome sequences. Numbers above the branches indicate the bootstrap support values.
